# Red light-emitting diode on skin healing: an *in vitro* and *in vivo* experimental study^☆^

**DOI:** 10.1016/j.abd.2024.02.008

**Published:** 2024-11-08

**Authors:** Tuany R. Schmidt, Belkiss C. Mármora, Fernanda T. Brochado, Lucas Gonçalves, Paloma S. Campos, Marcelo L. Lamers, Aurigena A. de Araújo, Caroline A.C.X. de Medeiros, Susana B. Ribeiro, Marco A.T. Martins, Emily F.S. Pilar, Manoela D. Martins, Vivian P. Wagner

**Affiliations:** a Department of Pathology, Faculty of Dentistry, Universidade Federal do Rio Grande do Sul, Porto Alegre, RS, Brazil; b Department of Pediatric Dentistry, Orthodontics and Public Health, Faculty of Dentistry, Universidade de São Paulo, Bauru, SP, Brazil; c Post-Graduation Program Oral Science/Post-Graduation Program in Pharmaceutical Science, Department of Biophysics and Pharmacology, Universidade Federal do Rio Grande do Norte, Natal, RN, Brazil; d Department of Biophysics and Pharmacology/ Postgraduate Program in Biological Science and Rede Nordeste de Biotecnologia, Universidade Federal do Rio Grande Norte, Natal, RN, Brazil; e Postgraduate Program in Biological Science and Rede Nordeste de Biotecnologia/Renorbio, Universidade Federal do Rio Grande Norte, Natal, RN, Brazil; f Department of Oral Medicine, Hospital de Clínicas de Porto Alegre, Universidade Federal do Rio Grande do Sul, Porto Alegre, RS, Brazil; g Experimental Pathology Unit, Hospital de Clínicas de Porto Alegre, Universidade Federal do Rio Grande do Sul, Porto Alegre, RS, Brazil; h Department of Oral Diagnosis, Faculty of Dentistry, Universidade de Campinas, Piracicaba, SP, Brazil; i Oral Medicine Department, Faculty of Dentistry, Universidade de São Paulo São Paulo, SP, Brazil

**Keywords:** Cell culture techniques, Cytokines, Dermatology, Low-level light therapy, Models, animal, Oxidative stress

## Abstract

**Background:**

The clinical advantages of light-emitting diode (LED) therapy in skin healing and its underlying mechanism remain subjects of ongoing debate.

**Objective:**

This study aims to explore the impact of LED therapy on normal skin keratinocytes (HaCaT) and in the repair of full-thickness dorsal wounds in Wistar rats.

**Methods:**

HaCaT cell viability (SRB assay) and migration (scratch assay) were assessed under LED therapy, comparing stress conditions (2.5% FBS) with sham irradiation and optimal conditions (10% FBS). In vivo, 50 rats with induced wounds were divided into Sham and LED (daily treatment) groups. Euthanasia occurred at 3, 5, 10, 14, and 21 days for clinical, morphological, oxidative stress (MDA, SOD, and GSH), and cytokine analyses (IL-1β, IL-10, TNF-α).

**Results:**

LED therapy significantly enhanced keratinocytes viability compared to sham irradiation, with minimal impact on cell migration. Clinical benefits were prominent on day 10, influencing inflammation progression and resolution on days 3 and 10. Re-epithelization remained unaffected. Reduced MDA and increased GSH levels were observed throughout, while SOD levels varied temporally. Notably, on day 10, LED significantly decreased IL-1β, IL-10, and TNF-α.

**Study limitations:**

Although translational, clinical trial confirmation of observed benefits is warranted.

**Conclusions:**

LED therapy expedites cutaneous healing in the experimental model, primarily modulating inflammation and enhancing antioxidant activity.

## Introduction

Skin wounds represent a burden in health care. Medicare cost projections for all wounds in the 2014 data set ranged from $28.1 to $96.8 billion.^1^ It is also estimated that nearly 2.5% of the total US population faces quality of life impairment associated with chronic wounds and this will probably rise in the following decades.^2^ A multitude of risk factors linked to chronic wounds persist or are on the ascent within society. These factors include an aging population, diabetes, obesity, and the emergence of antibiotic-resistant infections. It is imperative to identify therapies that are both safe and efficacious from a clinical standpoint. Likewise, comprehending the mechanisms by which these therapies operate at the cellular and tissue levels can provide valuable insights, enabling their application based on a sound biological rationale.

Photobiomodulation (PBM) is a well-established therapeutic approach to treat inflammatory conditions such as wounds.^3^ As for any other drug therapy, dosage, in this circumstance called dosimetry, is of paramount importance for the effectiveness of the therapy. It includes wavelength, power density, fluency, pulse, irradiated area (spot size), time, and number of sessions, among others. The studied group has previously shown that changes in energy density^4^ or wavelength and power^5^ influence cell and tissue response. Most of the studies developed assessing PBM in wound healing focused on protocols using red or near-infrared laser irradiation; showing promising results.^6^, ^7^, ^8^ There still appears to be a gap in the literature regarding the effects of red light-emitting diode (LED) irradiation.

LED and laser irradiation differ in light coherence and directionality. Other differences can be seen as advantages of LED and justify further research on it. For example, the typical larger spot size of LED devices often facilitates comprehensive irradiation of the entire wound area, ensuring effective coverage.^9^ LED devices are also considerably less expensive compared to lasers.^10^, ^11^ Based on these advantages and a lack of deep research in this area, the aim was to determine the effects of red LED therapy on cutaneous wound healing. Normal keratinocytes were used to assess the effects on cell proliferation and migration. The authors also investigated the impact at the clinical and tissue levels using full-thickness wound models. Key mechanisms such as oxidative stress and cytokine release were also examined.

## Material and methods

### LED irradiation

For both *in vitro* and *in vivo* models, irradiation groups received treatment with an LED device (Oncollux, Cosmedical, São Paulo, SP, Brazil) in a plate format measuring 6 × 3 × 0.6 cm and composed of 6 light emission points with a wavelength of 660 ± 20 nm and power of 5 Mw. Each irradiation lasted 7 minutes, providing an energy density of 2.7 J/cm^2^ of energy, 2 J per point.

### In vitro experiments

#### Cell culturing

The immortalized human keratinocyte cell line HaCaT (#T002000; AddexBio, San Diego, CA, US) was acquired at the Cells and Tissue Bank of Rio de Janeiro, Brazil. The cells were cultured in Dulbecco modified Eagle medium (DMEM-high glucose culture media; GIBCO, Thermo Fisher Scientific Inc., Waltham, MA, US), supplemented with 10% Fetal Bovine Serum (FBS) (GIBCO) and 1% penicillin-streptomycin (GIBCO). The cells were maintained at 37 °C, 95% relative humidity, and 5% CO_2_. All culturing procedures were performed under sterile conditions in a laminar flow hood (Thermo Scientific 1300 series A2).

#### Experimental group division

Three experimental groups were used:

Control sham-irradiation 10% (Sham 10%): Cells were not irradiated and were cultivated in a complete medium (DMEM) with 10% FBS.

Control sham-irradiation 2.5% (Sham 2.5%): Cells were not irradiated and were cultivated in nutrient-deficient DMEM with 2.5% FBS, simulating a stress condition.

LED: Cells were irradiated with LED and cultivated in nutrient-deficient DMEM with 2.5% FBS. The irradiation was performed in a dark environment, with the LED device placed in direct contact with the culture plates. To prevent any light scattering that could affect other groups, each experimental group was seeded in separate plates. The sham groups were subjected to the same procedures as the irradiated group, with the exception that the LED device remained turned off.

#### Cell viability assessment

Cell viability was determined using the Sulforhodamine B (SRB) assay as previously reported.^5^ Briefly HaCaT cells were seeded in a 96-well plate (Kasvi). After proper attachment, the culture medium for all groups (except Sham 10%) was changed to 2.5% FBS. After 2 hours, LED irradiation was performed, and 24 hours later the cells were subjected to the SRB assay. The absorbance was measured at a wavelength of 560 nm using a microplate reader (Synergy 2, BioTek Instruments, Inc). This assay was performed in sextuplicate.

#### Migration assay

Cell migration was assessed using the scratch (wound healing) assay. HaCaT cells were cultured in twelve-well plates until they reached confluence. Two hours before LED irradiation, the media for Sham 2.5% and LED groups were replaced with 2.5% FBS media. A p200 pipette tip was employed to create a wound on the cell monolayer by scraping two straight lines (vertical and horizontal), resulting in a cross-shaped wound. Cell debris was removed by PBS washes, and fresh media was added according to the experimental group. The LED group was irradiated once a day. Photographs of standard areas of the wound were taken at 0 h, 12 h, 24 h, 36 h, and 48 h time points using an inverted microscope. The ImageJ software (Fiji Version 1.44a, National Institutes of Health, Bethesda, MD, USA) was used to measure the open wound area at each time point. This assay was performed in triplicate.

### In vivo study

#### Animal model and experimental procedure

The present study received approval from the Ethics Committee on Animal Use of the Porto Alegre University Hospital (HCPA, Brazil) under protocol nº 2018-0624. All experiments were conducted following the guidelines outlined in the “Guide for the Care and Use of Laboratory Animals”. The animals were handled with humane treatment. The sample size was determined based on previous studies employing similar methodologies.^4^, ^5^ A total of 55 eight-week-old male rats (Rattus norvegicus albinus, Rodentia, Mammalia, Wistar lineage), weighing between 250 g to 300 g, were used. The rats were housed in groups of 2 to 4 animals under standard temperature conditions (20 ° to 24 °C) and a 12 -h light/dark cycle. They had ad libitum access to solid chow and water. For the wounding procedure, the animals were anesthetized using inhalable isoflurane, and the future wound area was shaved. Full-thickness circular wounds, 10 mm in diameter, were created on the animals' backs using a punch biopsy technique. To manage pain without influencing the inflammatory response, animals received two daily intraperitoneal doses of tramadol (20 mg/kg).

The animals were randomly allocated (based on body weight) into two groups: Sham Group (n = 25) and LED Group (n = 25). LED therapy commenced immediately after the surgical procedure and was performed daily until the predetermined euthanasia days. The LED device was placed in contact with the skin ulcer, while animals in the Sham group received identical handling without LED irradiation. On days 3, 5, 10, 14, and 21, five rats from each group were euthanized using inhalant isoflurane anesthesia. The back injuries were photographed, removed, and the specimens were processed for histopathological study and analysis of cytokines and antioxidant activity.

#### Clinical assessment

The wounds were documented alongside a reference object with pre-established measurements (1 cm by 1 cm). A blinded assessor quantified the wound area in pixels using the ImageJ 1.48v software (National Institutes of Health, USA). Subsequently, the values were converted to square millimeters (mm^2^) using the reference object as a scaling standard.

#### Histopathological evaluation

Samples preserved in 10% formalin were further processed for embedding in paraffin following a conventional protocol. Sections of 5 µm thickness were cut and then stained with hematoxylin and eosin for histopathological analysis. Two experienced pathologists conducted a semi-quantitative assessment of reepithelization and inflammation, arriving at a consensus-based final score.^4^ The evaluations were performed in a blinded manner.

Reepithelization scores were defined as follows:

Grade 0: No reepithelization.

Grade 1: Reepithelization covering less than half of the wound.

Grade 2: Reepithelization covering more than half of the wound.

Grade 3: Reepithelization covering the entire wound with irregular thickness.

Grade 4: Reepithelization covering the entire wound with a normal thickness.

Inflammatory process scores included:

Grade 1: Acute inflammation (pyogenic membrane at the superficial wound area without vascular events in the underlying connective tissue).

Grade 2: Predominance of diffuse acute inflammation (vascular phenomena such as edema and hyperemia predominate).

Grade 3: Predominance of a chronic inflammatory process (presence of inflammatory cells, angiogenesis, and fibroplasia).

Grade 4: Resolution and healing (reduction or disappearance of chronic inflammation).

#### Redox analysis

Redox status was assessed by measuring the levels of Malonaldehyde (MDA), Glutathione (GSH), and Superoxide Dismutase (SOD) content as previously described.^5^ MDA is a byproduct of lipid peroxidation, and higher MDA levels indicate increased oxidative activity, while GSH and SOD levels were evaluated to determine antioxidant activity. Results were expressed in nanomoles of MDA per gram of tissue, GSH units per milligram of tissue, and *U* per gram of protein for SOD content.

#### Cytokine immunoassays

Cytokine levels were quantified following the manufacturer's instructions. Briefly, skin samples were homogenized and processed. Interleukin (IL)-1β, IL-10, and Tumor Necrosis Factor (TNF)-α levels were determined using commercial enzyme-linked immunosorbent assay kits (R & D Systems, Minneapolis, MN). The results were expressed in picograms per milliliter (pg/mL).

### Statistical analysis

In vitro and in vivo data were analyzed using GraphPad Prism 8.0 statistical software (GraphPad Software, San Diego, CA, USA). Group comparisons were performed using one-way analysis of variance (ANOVA). Post-hoc Bonferroni tests were employed to ascertain the significance of differences between the experimental groups. All data were presented as mean and Standard Deviation (±SD). A p-value below 0.05 was considered indicative of statistical significance, denoted by asterisks (*p < 0.05; **p < 0.01; ***p < 0.001; ****p < 0.0001).

## Results

### LED therapy increases keratinocyte viability in vitro

**Figure 1:**
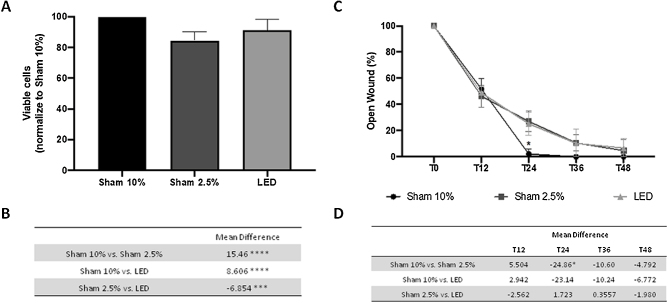
LED effects on human keratinocytes viability and migration *in vitro*. (A) Percentage of viable cells based on absorbance of SRB assay normalized to Sham 10% group. (B) Main difference in cell viability between experimental groups. (C) Percentage of open wound normalized to baseline (T0) analysis in different experimental times. (D) Main difference in cell migration between experimental groups throw-out the experimental times. Asterisks denote significant results.

The authors compared HaCat cell's viability under optimal (Sham 10%) and stress condition (Sham 2.5%) with LED irradiation under the same stress conditions, which stimulates the healing environment stress and allow PBMT effect. The authors observed that after one irradiation LED therapy promoted increased cell viability compared to the Sham 2.5% group (p < 0.001) (Fig. 1A and B). Yet, even having a significant impact, LED therapy was not capable of restoring cell viability to its baseline value under optimal conditions (Sham 10% group, p < 0.0001).

### LED therapy has no significant impact on keratinocyte migration in vitro

Cell migration was evaluated through scratch assay. Stress conditions (2.5% of FBS) only impaired cell migration at T24 compared to the Sham 10% group (p < 0.05) (Fig. 1C and D). At this same moment, HaCaT cells treated with LED presented a wound area similar to both control groups (Sham 10% and Sham 2.5%) (Fig. 1C and D). This result suggests a limited effect of LED therapy in keratinocyte migration. On all other evaluation times, all groups presented similar values statistically.

### LED therapy accelerates clinical wound closure at an intermediate time point

**Figure 2:**
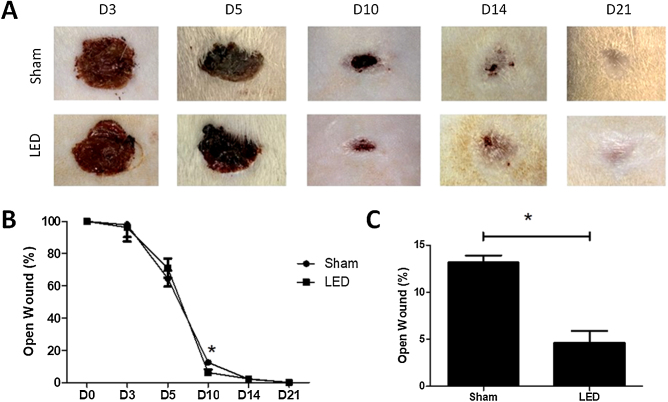
Effect of LED therapy on cutaneous healing of Wistar rats. (A) Representative clinical images the healing process of full thickness induced wounds according to experimental groups during the different analysis periods. Note that on day 10 the LED group presented a significantly smaller wound area compared to the Sham group. (B) Percentage of wound closure in different experimental times normalized to baseline wound area (day 0). (B) Main and SD of percentage of wound closure on day 10 in Sham (13.16 ± 1.50) and LED (4.62 ± 2.53) groups. Asterisks denote significant results.

Clinical effects of LED therapy on cutaneous healing were assessed on days 3, 5, 10, 14 and 21. Only at day 10, the wound area in the LED group was significantly smaller compared to the Sham group (p < 0.05) (Fig. 2A and B). On this day, while the Sham group had an average of 13.16% (±1.50) of the open wound area, animals treated with LED had only an average of 4.62% (±2.53) (Fig. 2C). On other experimental times, both groups had a similar percentage of wound closure (Fig. 2B).

### LED therapy modulates the inflammatory process during cutaneous wound healing in vivo but has no impact on re-epithelization

**Figure 3:**
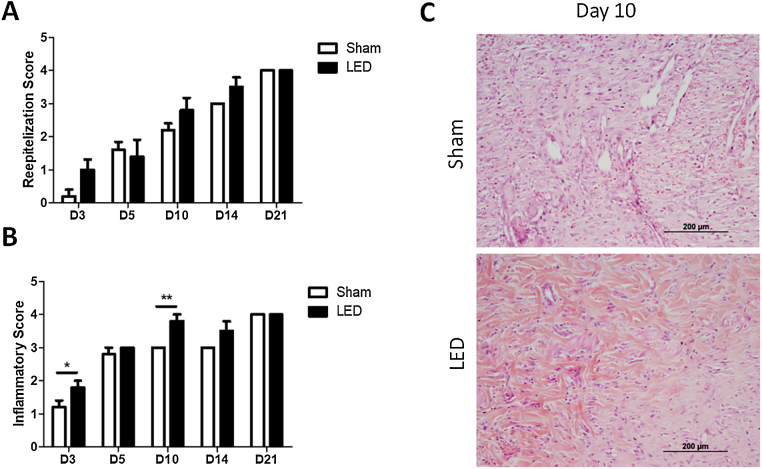
Histopathological evaluation of cutaneous healing. (A) Main (±SD) scores of reepithelization according to experimental groups during the different analysis periods. (B) Main (±SD) scores of inflammations according to experimental groups during the different analysis periods. Asterisks denote significant results. (C) Wound healing site in Sham and LED groups on day 10. Note the abundance of collagen deposition and increase in angiogenesis in LED-treated wounds. (Hematoxylin & Eosin stain, ×100).

The authors further evaluated how LED therapy influences cutaneous tissue healing by morphological analysis of reepithelization and inflammation. No significant differences were observed for reepithelization scores in any of the experimental periods (Fig. 3A). Yet, a tendency of a more advanced reepithelization process (higher score) was noted in the LED group on days 3, 10 and 14 (Fig. 3A).

LED therapy had an impact on modulating the inflammatory process. Animals treated with LED presented significantly higher inflammatory scores, which represent a more advanced healing process, on days 3 and 10 (Fig. 3B). LED therapy accelerated the inflammatory process chronification and resolution, as can be evidenced on photomicrographs on day 10 in which wounds from the Sham group still present inflammatory cells and collagen deposition was less evident while wounds irradiated with LED present increased collagen deposition, evident angiogenesis and less intense inflammatory infiltrate (Fig. 3C).

### LED therapy triggers anti-oxidative activity and modulates inflammatory cytokines release during cutaneous wound healing in vivo

**Figure 4:**
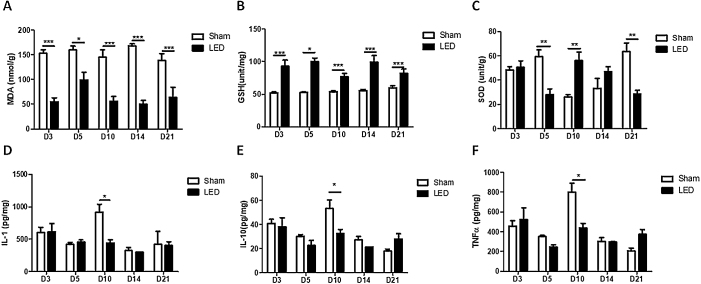
Effect of LED therapy on REDOX state and inflammatory cytokines. (A) Main (±SD) levels of (A) MDA, (B) GSH, (C) SOD, (D) IL-1β, (E) IL-10, and (F) TNF-α according to experimental groups during the different analysis periods. Asterisks denote significant results.

LED induced an anti-oxidative effect on all experimental times, as evidenced by significantly lower levels of MDA (oxidative marker) and higher levels of GSH (anti-oxidative enzyme) in the LED group compared to the Sham group (Fig. 4A and B). The effects of LED therapy on SOD levels (anti-oxidative enzyme) were not constant: LED induced an increase in SOD levels on day 10 (corroborating with the other markers) but had an intriguing inhibitory effect on days 5 and 21 (Fig. 4C).

Significant effects of LED therapy on inflammatory cytokines occurred only on day 10. At this experimental time, LED induced a decrease in the levels of all cytokines evaluated: IL-1β, IL-10 and TNF-α (Fig. 4D, E and F). On the other experimental times, the effects were variable, showing a tendency of increase or decrease depending on the cytokine/time evaluated, but with no significant differences between experimental groups.

## Discussion

Inadequate management of skin wounds can have far-reaching consequences, including prolonged healing times, an elevated risk of recurrence or infection, and potential complications that may necessitate hospitalization.^12^ Numerous therapeutic approaches have been explored with the goal of expediting wound closure. In this study, the authors delved into the effects of LED therapy on skin healing. The present findings underscored several strengths of LED therapy, including its ability to modulate the redox state. However, the authors also uncovered certain limitations associated with this therapeutic approach, such as no impact on keratinocyte migration.

The skin acts as a protective barrier against microorganisms, with keratinocytes safeguarding the connective tissue.^13^ Rapidly restoring the keratinocyte layer lost in skin ulcers, known as reepithelization, is crucial to prevent further infection. The present study found that LED influenced keratinocyte proliferation but not cell migration. This is in contrast with the findings by Sutterby et al., (2022), who demonstrated that red LED (660 nm) irradiation promoted HaCat proliferation and migration. For the scratch assay, the authors performed a single 10-min LED exposure (0.8 mW, 0.3 J/cm^2^). Significant differences between LED and the control group were observed at 48 h and 72 h.^9^ The disparity in outcomes could be attributed, in part, to differences in dosimetry parameters. Notably, the dose used in their study was significantly lower than ours. Previous research has shown that lower doses of red laser irradiation (4 J/cm^2^) can have more pronounced effects compared to higher doses (20 J/cm^2^).^4^ However, various methodological factors, such as wound size, culture conditions, and baseline metabolic activity, may also have played a role in these varying results. To mitigate the potential influence of cell proliferation during the scratch assay, it is advisable to implement serum starvation conditions (as the authors did) or induce cell cycle arrest using pharmacological agents.^14^ Unfortunately, it is unclear whether Sutterby et al. employed any of these methods, leaving room for the possibility that the closure of the gap area observed in their study could, in part, be attributed to the effect of LED therapy on cell proliferation. Other conditions that have been tested in PBM research include IL-4- or TNF-α and IFN-γ-conditioned media to mimic an inflammatory milieu. PBM with a red laser has been shown to increase HaCaT proliferation in a scratch assay under these conditions.^15^

The *in vivo* assessment also showed no significant impact of LED irradiation on re-epithelization scores, consistent with the in vitro results. It can be concluded that LED has limited efficacy in expediting epithelial barrier closure in the parameters used herein. The clinical advantages of LED therapy in the red spectrum appear to be associated with its capacity to modulate the inflammatory process and other mechanisms, as evidenced in the present study. At day 10, the histological examination clearly indicated a marked increase in collagen deposition in the LED-treated group compared to the control group. This observation aligns with the findings of Kim et al. (2015), who demonstrated that red LED irradiation at 660 nm heightened the metabolic activity of fibroblasts.^16^ The clinical benefits of LED therapy in a murine model observed herein align with prior animal studies.^17^, ^18^ The primary clinical outcome was would size and no quantitative or qualitative assessment of the scar tissue was performed, which represents a limitation. Scarring poses notable aesthetic and functional implications for patients and previous studies have shown positive effects of LED-based therapy, particularly at high energy doses, on scar observer rating.^19^ Further studies confirming the positive effect of LED therapy on scaring are needed.

As far as the authors are concerned, this study marks the first experimental exploration of LED therapy's impact on REDOX and cytokine release at the tissue level during skin healing.^3^ On day 10, LED therapy significantly reduced levels of IL-1β, IL-10, and TNF-α. At this juncture, this suggests that LED has the capacity to attenuate the pro-inflammatory acute phase and facilitate the progression of the proliferative phase. Notably, the authors observed the ability to modulate oxidative stress throughout the experimental periods, fostering an anti-oxidative environment through MDA decrease and GSH release. These observations align with in vitro studies investigating the impact of red LED irradiation on oxidative stress. For instance, LED irradiation at 625 nm for 60 minutes significantly decreased intracellular Reactive Oxygen Species (ROS) in HaCat cells, as measured by 2′, 7′-Dichlorofluorescein Diacetate (DCF-DA) fluorescence.^20^ Similar reductions in ROS following red LED exposure (630–670 nm) were noted in pulp fibroblasts^21^ and Muller glial cells.^22^ Ulcers in diabetic patients are primarily associated with oxidative stress and the release of pro-inflammatory cytokines.^23^ The innovative findings position LED irradiation as a promising therapeutic approach for diabetic wounds. Future investigations focusing on the use of LED irradiation in diabetic animal models hold clear translational value and are warranted.

As the body of research exploring the benefits of red and infrared laser therapy for wound healing, including diabetic ulcers,^24^ continues to expand, the potential of LED therapy remains underappreciated. The present study has unveiled significant biological responses that strongly advocate for the adoption of LED therapy. Furthermore, it is worth emphasizing that LED devices offer additional advantages, such as cost-effectiveness. For instance, the cost per milliwatt (mW) of LED devices is, on average, one hundred times lower compared to lasers.^11^ This affordability facilitates the acquisition of these devices by clinics and hospitals or even patients, enhancing accessibility, especially in low-income countries. The convenience of the application is another key factor. LED devices can emit light over a broader surface area due to their wider spectral bandwidth.^9^ This feature is particularly advantageous for treating skin wounds, which can often be substantial, especially in the context of diabetic foot ulcers. As a matter of fact, positive clinical results on diabetic foot ulcers of red-LED devices that can be safely used at home and handled by the patient have been published, confirming the hypothesis.^25^

In conclusion, LED therapy under the specific conditions tested here significantly expedited the process of cutaneous healing *in vivo* when compared to sham irradiation. The clinical benefits appear to be attributed to the LED's capacity to modulate the release of inflammatory cytokines, leading to a notable reduction in pro-inflammatory signals by day 10. Simultaneously, it fostered an antioxidative environment throughout the entire healing trajectory. On the other hand, the present research findings indicate that the LED protocol tested had a limited impact on keratinocyte migration, a conclusion supported by both *in vitro* and *in vivo* data analysis.

## Financial support

This study was funded by the Research Group of Hospital de Clínicas de Porto Alegre (DIPE/HCPA: 2018-0624) and Azena Medical, which provided laser equipment and research funding. The funders had no role in study design, data collection, and analysis, decision to publish, or preparation of the paper.

## Authors’ contributions

Tuany Rafaeli Schmidt: Data collection, analysis and interpretation; writing the original draft.

Belkiss Câmara Mármora: Study concept and design; data collection, analysis and interpretation; writing-reviewing and editing.

Fernanda Thomé Brochado: Data collection and analysis; writing-reviewing and editing.

Lucas Gonçalves: Data collection and analysis; writing-reviewing and editing.

Paloma Santos Campos: Data collection and analysis; writing-reviewing and editing.

Marcelo Lazzaron Lamers: Data analysis and interpretation; writing-reviewing and editing.

Aurigena Antunes de Araújo; Data analysis and interpretation; writing-reviewing and editing.

Caroline Addison Carvalho Xavier de Medeiros: Data analysis and interpretation; writing-reviewing and editing.

Susana Barbosa Ribeiro: Data analysis and interpretation; writing-reviewing and editing.

Marco Antonio Trevizani Martins: Data analysis and interpretation; writing-reviewing and editing.

Emily Ferreira Salles Pilar: Data analysis and interpretation; writing-reviewing and editing.

Manoela Domingues Martins: Study concept and design; data analysis and interpretation; funding acquisition; supervision; writing-reviewing and editing.

Vivian Petersen Wagner: Study concept and design; data analysis and interpretation; statistical analysis; writing the original draft.

## Conflicts of interest

None declared.
